# Interaction Between Vascular Endothelial Growth Factor Gene Polymorphism and Smoking on Gastric Cancer Risk in Chinese Han Population

**DOI:** 10.3389/pore.2022.1610495

**Published:** 2022-08-25

**Authors:** Longyue Wang, Shuaishuai Xiao, Yiming Zheng, Zefeng Gao

**Affiliations:** Department of General Surgery Two, Shanxi Province Cancer Hospital, Shanxi Hospital Affiliated to Cancer Hospital, Chinese Academy of Medical Sciences, Cancer Hospital Affiliated to Shanxi Medical University, Taiyuan, China

**Keywords:** gastric cancer, smoking, interaction, single nucleotide polymorphisms, vascular endothelial growth factor

## Abstract

**Aim:** In this study, we aimed to evaluate the associations of vascular endothelial growth factor (*VEGF*) gene single nucleotide polymorphisms (SNPs) and its interaction with current smoking with gastric cancer (GC) risk in the Chinese Han population.

**Methods:** We used logistic regression model to test the association between *VEGF* gene polymorphism and the risk of GC. The association strength was evaluated by odds ratio (OR) and 95% confidence interval (CI) calculated using logistic regression. Generalized multifactor dimensionality reduction (GMDR) was used to analyze the effect of the interaction between *VEGF* gene and current smoking on GC risk.

**Results:** Logistic regression analysis showed that the risk of GC was significantly higher in rs10434 -G allele carriers than that in AA genotype carriers (AG + GG and AA), and the adjusted OR (95% CI) = 1.64 (1.24–2.08). In addition, we found a significantly higher GC risk in subjects with rs833061-T allele than those with CC allele (CT + TT and CC), adjusted or (95% CI) = 1.43 (1.10–1.87). We also found a statistically significant two- locus model (*p* = 0.018), including rs10434 and current smoking, indicating a significant interaction between rs10434 and current smoking on the risk of GC. Hierarchical analysis found that current smokers with AG or GG genotype have the highest GC risk, compared to never- smokers with AA genotype, OR (95% CI) = 2.43 (1.64–3.28).

**Conclusion:** We found that rs10434 -G and rs833061-T alleles, gene- environment interaction between rs10434, and current smoking were all related to increased GC risk.

## Introduction

Gastric cancer (GC) is a common cancer type globally. Newly diagnosed gastric cancer account for 5.7% of all cancers, with over a million cases a year. The high incidence rate and high mortality rate make GC one of the most important cancers threatening human health [1, 2]. GC is the third leading cause of cancer-related death. The incidence rate differs depending on country, and has a higher incidence in East Asia, Eastern Europe, and South America. But the incidence rate is relatively low in Oceania, Africa, and North America [3]. Previous studies have reported some risk factors for GC, the most common of which include Helicobacter pylori infection, tobacco smoking, low fiber intake, high salt or smoked food intake, and genetic factors [4].

Vascular endothelial growth factor (VEGF) is an effective mitogen in the endothelium. Its main role is to regulate angiogenesis and postnatal vascular remodeling. Its expression is up- regulated under various pathophysiological conditions [5]. Therefore, vascular endothelial growth factor is a lymphangiogenic growth factor, which plays an important role in tumor lymphangiogenesis by activating *VEGF* receptor [6]. *VEGF* gene is located on chromosome 6p21.1 and contains 9 exons [7]. Previously, several studies have reported the relationship between *VEGF* gene single nucleotide polymorphism (SNP) and several cancer’s risk, including bladder cancer [8], lung cancer [9], and renal cell carcinoma [10]. However, the etiology of the relationship between *VEGF* gene SNPs and GC risk is very complicated and yet to be completely understood. In addition, the pathogenesis of GC involves not only genetic factors but also environmental factors; no study has focused on the synergistic effect between *VEGF* gene and environmental factors. Therefore, the main purpose of this study is to evaluate whether there is a statistical association between *VEGF* gene polymorphism and GC risk in the Chinese Han population, and the impact of the interaction between *VEGF* gene and environmental lifestyle factors on GC risk.

## Methods

### Study Population

All participants in this study were recruited from Shanxi cancer hospital from June 2016 to July 2020. Those with malignant diseases other than gastric cancer, such as cardiovascular disease and other tumors, were not included in the case group. The control group was randomly selected from healthy individuals of a chronic non-communicable disease screening program in the area. The control group was finally selected by matching the subjects of the case group by 2:1 according to age and gender. Consequently, a total of 1,451 eligible participants were included in the study; the mean age of all participants were 64.0 ± 10.1 years. A total of 483 eligible gastric cancer patients were included in the case group and 968 healthy participants were included in the control group. We designed a simple questionnaire to collect participants’ general demographic information and clinical and biochemical index data. Whole blood samples were collected from each participant. Written informed consent was obtained from all participants.

### Genotyping Methods

A total of four SNPs within *VEGF* gene were selected, including rs10434, rs3025039, rs2010963, and rs833061. We collected 3 ml blood samples from all participants, and these blood samples were treated with EDTA and stored in a −20°C refrigerator. The subject’s DNA was extracted according to the instructions of the DNA blood micro- Kit (Qiagen, Hilden, Germany). In this study, genotypes of four selected SNPs were determined by using the polymerase chain reaction-restriction fragment length polymorphism (PCR-RFLP) assay as previously described [11, 12]. The product was digested with restriction enzymes, which were shown in Table 1, and analyzed onto 2% agarose gel. All staff involved in genotype testing were blinded to the basic information and phenotypes of all participants and, after the experiment, we repeated the test on a randomly selected 10% of the samples to ensure the accuracy and reproducibility of the test results. Repeat test results are 100% consistent with the first test.

**TABLE 1:** Description and primer sequences used for genotyping for 4 SNPs within *VEGF* gene.

SNP ID	Chromosome	Location	Nucleotide substitution	Restriction enzymes	Primer sequences
rs3025039*+936C/T*	6:43784799	Intron variant	C>T	*NlaIII*	F: 5′- ACA​CCA​TCA​CCA​TCG​ACA​GA-3′
					R: 5′- GGC​TCG​GTG​ATT​TAG​CA-3′
rs2010963*-634G/C*	6:43770613	Intron variant	G > C	*BsmFI*	F:5′-TTGCTTGCCATTCCCCACTTGA-3′
					R: 5′- CCG​AAG​CGA​GAA​CAG​CCC​AGA-3′
rs10434 *+1612G/A*	6:43753212	Intron variant	A> G	*MnlI*	F:5′-TTCGCT TACTCTCACCTGCTT C-3′
					R: 5′- GCT​GTC​ATG​GGC​TGC​TTC​T-3′
rs833061*-460C/T*	6:43769749	Intron variant	C>T	*BsaHI*	F:5′-TGAGTGTGTGCGTGTGGGGTTGAGCG-3′
					R: 5′- AGA​GCC​GTT​CCC​TCT​TTG​CTA​G-3′

### Statistical Analysis

All data were tested using SPSS 22.2 software, and we calculated the mean and standard deviation (SD) for continuous variables conforming to normal distribution and calculated the percentage for classified variables. The χ^2^ testing was used for comparison of categorical variables, and the T-test was used to compare the mean and SD. The online software (SNPStats: https://www.snpstats.net/) was used for analysis on association between four SNPs and GC risk. Generalized multifactor dimensionality reduction (GMDR) [13] was employed to test the interaction between the four SNPs and current smoking. The consistency of cross validation and the accuracy of test balance and symbolic test were calculated to evaluate the interaction of each selection.

## Results

A total of 1,451 participants with an average age of 64.0 ± 10.1 years were selected, made up of 483 GC patients and 968 healthy controls. Table 2 shows the general demographic characteristics and clinical indicators in GC patients and controls. The mean age, BMI, and percentage of males were not significantly different between GC patients and controls. The percentage for current smoking and alcohol drinking were significantly higher in GC patients than in controls. In addition, 67.7% of the GC patients were non-cardia and the tumor size of 81.2% GC patients was ≤5 cm.

**TABLE 2:** General characteristics of GC patients and controls.

Variables	GC patients (N = 483)	Controls (N = 968)	*p-*values
Gender, N (%)			0.824
Males	311 (64.4)	629 (65.0)	
Females	172 (35.6)	339 (35.0)	
Age (year) (Means ± SD)	63.4 ± 12.7	64.5 ± 13.1	0.128
Smoking, N (%)			0.010
Current smokers	159 (32.9)	256 (26.4)	
Never smokers	324 (67.1)	712 (73.6)	
Alcohol drinkers, N (%)			0.006
Current drinkers	178 (36.8)	287 (29.6)	
Never drinkers	305 (63.2)	681 (70.4)	
BMI (kg/m^2^) (Means ± SD)	23.9 ± 9.3	24.4 ± 9.8	0.352
Tumor location, N (%)			
Non-Cardia	327 (67.7)		
Cardia	156 (32.3)		
Tumor size, N (%)			
≤5 cm	392 (81.2)		
>5 cm	91 (18.8)		

The genotype distribution in the control group was consistent with the HWE (All *p*- values more than 0.05). The allele frequencies of rs10434 -G and rs833061-T were significantly higher in GC patients than that in control group (30.0% and 20.0%, 28.9% and 20.7%, respectively). Logistic regression analysis showed that the risk of GC was significantly higher in rs10434 -G allele carriers than that in AA genotype carriers (AG + GG and AA), and the adjusted OR (95% CI) = 1.64 (1.24–2.08). In addition, we found a significantly higher GC risk in subjects with rs833061-T allele than those with CC allele (CT + TT and CC), adjusted or (95% CI) = 1.43 (1.10–1.87) (Table 3).

**TABLE 3:** Logistic regression analysis on association between four *VEGF*–SNPs and GC risk.

SNPs	Genotypes and alleles	Frequencies N (%)		OR (95%CI)^a^	*p*-values
		Controls (*n* = 968)	Cases (*n* = 483)		
rs3025039 (*+936C/T*)					
	CC	562 (58.1)	257 (53.2)	Ref	
	CT	348 (35.9)	188 (38.9)	1.25 (0.87–1.65)	
	TT	58 (6.0)	38 (7.9)	1.33 (0.79–1.90)	
	C	1,472 (76.0)	702 (72.7)	Ref	
	T	464 (24.0)	264 (27.3)	1.28 (0.85–1.79)	
HWE test for controls					0.672
rs10434 (*1612G/A*)					
	AA	627 (64.8)	243 (50.3)	Ref	
	AG	294 (30.4)	190 (39.3)	1.54 (1.17–1.94)	
	GG	47 (4.8)	50 (10.4)	2.03 (1.49–2.59)	
	A	1,548 (80.0)	676 (70.0)	Ref	
	G	388 (20.0)	290 (30.0)	1.64 (1.24–2.08)	
HWE test for controls					0.103
rs2010963 (*-634G/C*)					
	GG	562 (58.1)	249 (51.6)	Ref	
	GC	345 (35.6)	189 (39.1)	1.21 (0.89–1.56)	
	CC	61 (6.3)	45 (9.3)	1.42 (0.80–2.07)	
	G	1,469 (75.9)	687 (71.1)	Ref	
	C	467 (24.1)	279 (28.9)	1.24 (0.87–1.69)	
HWE test for controls					0.412
rs833061 (*-460C/T*)					
	CC	617 (63.7)	249 (51.6)	Ref	
	CT	302 (31.2)	189 (39.1)	1.40 (1.12–1.73)	
	TT	49 (5.1)	45 (9.3)	1.58 (0.97–2.22)	
	C	1,536 (79.3)	687 (71.1)	Ref	
	T	40 (20.7)	279 (28.9)	1.43 (1.10–1.87)	
HWE test for controls					0.132

a Adjusted for gender, age, smoking, and alcohol consumption status, BMI.

The GMDR model was used to evaluate the synergy effect between four *VEGF* gene SNPs and current smoking on the susceptibility to GC (Table 4). We found a statistically significant two- locus model (*p* = 0.018), including rs10434 and current smoking, indicating a significant interaction between rs10434 and current smoking on the risk of GC. The cross- validation consistency of the two- locus model was 10/10, the test accuracy was 0.632, and *p*- value was 0.018. To obtain the odds ratios and 95%CI for the joint effects of rs10434 polymorphism and current smoking on GC susceptibility, we conducted hierarchical analysis for interaction between rs10434 and current smoking on GC risk by using logistic regression. We found that current smokers with AG or GG genotype have the highest GC risk, compared to never- smokers with AA genotype, OR (95%CI) = 2.43 (1.64–3.28), after adjustment for gender, age, alcohol consumption status, and BMI (Table 5).

**TABLE 4:** GMDR analysis on the best interaction models between *VEGF* gene and current smoking.

Locus no.	Best combination	Cross-validation consistency	Testing balanced accuracy	*p-values* ^a^
**2**	1 * current smoking	**10/10**	**0.632**	**0.018**
3	1 * 4 * current smoking	8/10	0.601	0.182
4	1 * 4 * 2 * current smoking	7/10	0.532	0.256
5	1 * 4 * 2 * 3 * current smoking	6/10	0.524	0.425

a Adjusted for gender, age, alcohol consumption status, BMI.

The values in bold presented statistical differences (*p* < 0.05).

1-4 represent rs10434, rs3025039, rs2010963 and rs833061.

**TABLE 5:** Hierarchical analysis for interaction between rs10434 and current smoking on GC risk by using logistic regression.

rs10434	Current smoking	OR (95% CI)^a^	*p*-values
AA	No	1.00	—
AG or GG	No	1.36 (1.09–1.67)	0.031
AA	Yes	1.58 (1.16–2.03)	0.001
AG or GG	Yes	2.43 (1.64–3.28)	<0.001

a Adjusted for gender, age, alcohol consumption status, BMI.

## Discussion

In our study, we investigated the relationship of four SNPs of *VEGF* gene with GC risk. The results indicated that the risk of GC was significantly higher in rs10434 -G allele carriers than that in AA genotype carriers. In addition, we also found a significantly higher GC risk in subjects with rs833061-T allele than those with CC allele. Nevertheless, after several covariates’ adjustment, we found that no statistically significant correlation existed between rs3025039, rs2010963, and GC risk. Previously, several studies [14–19] focused on the association between SNPs of *VEGF* gene and GC risk in different populations. According to the research of Zhou et al [14] in the Chinese Han population, *VEGF* rs10434 (*+ 1612 G/A*) gene polymorphism may be related to a higher risk of gastric cancer, and the difference of genotype distribution may be related to Lauren classification and the location of gastric cancer, but they also found that rs2010963 and rs833061 were not associated with GC risk. In a Japanese population, Tahara et al [15] suggested that the *G1612A* mutation in *VEGF* gene was statistically associated with the risk of GC, but the association between *C936T* polymorphism and GC susceptibility was not statistically significant. A meta-analysis by Hong et al [16] including these studies showed that the 1612a allele of *VEGF* gene is a recessive susceptibility site of gastric cancer, with a 60% increased risk. However, the meta-analysis results of Zhuang et al [17] showed that people carrying *VEGF -634 G* allele may increase the risk of gastric cancer, while *VEGF +1612 G/A* G allele may reduce the risk of gastric cancer. No association was found between *+ 936C/T* and *- 460C/T* polymorphisms and susceptibility to gastric cancer. Liu et al. [18] conducted meta-analysis on Asian, European, and American populations respectively, and found that in the overall population, *VEGF-634 G> C* GG genotype was associated with the reduction of gastric cancer risk, *VEGF- 634 G> C*- C allele and GG genotype were associated with the risk in Caucasian people, while in the Asian population, *VEGF + 1612G/A* locus was significantly associated with the risk of GC. In our study, we also verified a significant relationship of rs10434 and rs833061 with GC susceptibility in the Chinese population. The mechanisms underlying the association between the *VEGF* gene and gastric cancer are not exhaustive. Previous studies have suggested that VEGF is one of the most important growth factors in the process of lymphatic angiogenesis and can play an important role in the progression of lymphangiogenesis in several tumors by activating the *VEGF* receptor. In the stomach, enhanced *VEGF* gene expression has been identified as contributing to the healing of GI lesions [20, 21]. In addition, gastric adenocarcinomas frequently show high levels of VEGF expression accompanied by an increase in intratumoral microvessel density. Higher levels of VEGF expression can also be observed in pre-cancerous gastric lesions, such as precancerous lesions, chronic atrophic gastritis, and intestinal chemosis [22].

GC susceptibility was influenced by many risk factors, including environmental and genetic factors, and the synergistic effect between gene and lifestyle factors [23]. Previously, the association between smoking habit and GC risk has been reported [24–27], and to date, no study focused on the effect of *VEGF* gene- smoking interaction on GC risk. In the current study, GMDR software was employed to investigate the interaction among four SNPs of *VEGF* gene and current smoking. We found a significant interaction between rs10434 and current smoking on the risk of GC; current smokers with AG or GG genotype have the highest GC risk, compared to never- smokers with AA genotype. The biological mechanism of the interaction between *VEGF* gene and smoking on the risk of gastric cancer is not very clear. Previous studies [27, 28] have confirmed that *VEGF* gene and smoking are related to the inflammatory response in the human body. Perhaps there is the same biological mechanism between them, resulting in a significant change to susceptibility of gastric cancer when they exist at the same time.

Some limitations existed in this study and should be explained. Firstly, there are too many SNPs in *VEGF* gene. In this study, only four SNPs are selected. These four SNPs could not represent all *VEGF* genes, but these SNPs are often mentioned in previous studies, and the results are different. Secondly, in this study, all environmental factors listed in Table 2 were included in GMDR software, but just the significant results were listed in Table 4; in the future, more lifestyle factors should be included in the interaction analysis. Lastly, we did not collect information about Helicobacter pylori infection, so the bias due to H. pylori infection may exist in this study.

In conclusion, our research shows rs10434 -G and rs833061-T alleles, gene- environment interaction between rs10434, and current smoking were all related to increased GC risk. In the future, more functional experiments are needed to verify the results obtained in our study.

## Data Availability

The datasets generated and/or analyzed during the current study are available from the corresponding author on reasonable request.
